# Micro/Nano Gas Sensors: A New Strategy Towards *In-Situ* Wafer-Level Fabrication of High-Performance Gas Sensing Chips

**DOI:** 10.1038/srep10507

**Published:** 2015-05-22

**Authors:** Lei Xu, Zhengfei Dai, Guotao Duan, Lianfeng Guo, Yi Wang, Hong Zhou, Yanxiang Liu, Weiping Cai, Yuelin Wang, Tie Li

**Affiliations:** 1 Science and Technology on Micro-system Laboratory, Shanghai Institute of Microsystem and Information Technology, Chinese Academy of Sciences, Shanghai, 200050, China; 2 Key Lab of Materials Physics, Anhui Key lab of Nanomaterials and Nanotechnology, Institute of Solid State Physics, Chinese Academy of Sciences, Hefei, 230031, Anhui, China; 3 California Institute of Technology, Pasadena, California 91125, USA

## Abstract

Nano-structured gas sensing materials, in particular nanoparticles, nanotubes, and nanowires, enable high sensitivity at a ppb level for gas sensors. For practical applications, it is highly desirable to be able to manufacture such gas sensors in batch and at low cost. We present here a strategy of *in-situ* wafer-level fabrication of the high-performance micro/nano gas sensing chips by naturally integrating microhotplatform (MHP) with nanopore array (NPA). By introducing colloidal crystal template, a wafer-level ordered homogenous SnO_2_ NPA is synthesized *in-situ* on a 4-inch MHP wafer, able to produce thousands of gas sensing units in one batch. The integration of micromachining process and nanofabrication process endues micro/nano gas sensing chips at low cost, high throughput, and with high sensitivity (down to ~20 ppb), fast response time (down to ~1 s), and low power consumption (down to ~30 mW). The proposed strategy of integrating MHP with NPA represents a versatile approach for *in-situ* wafer-level fabrication of high-performance micro/nano gas sensors for real industrial applications.

With the ever-increasing demands in applications and ever-growing expansion of application fields, more challenging specifications have been imposed on today’s gas sensors: high sensitivity, low power consumption, fast response time, and low cost. Nanostructured materials offer extremely high surface-to-volume ratios, high surface activities, and high carrier mobility^1^,^2^,^3^. Due to these distinct advantages, much effort has geared towards the fabrication process of such nanostructured gas sensing materials. Many nanostructured materials such as nanoparticles^4^,^5^, nanotubes^6^,^7^,^8^,^9^,^10^, nanowires^11^,^12^,^13^,^14^,^15^,^16^,^17^,^18^,^19^, and hollow spheres^20^,^21^,^22^ have been extensively researched for various gas sensing applications. In particular, nanostructured materials of metal oxide semiconductor (MOS), which possess remarkably high sensitivity and a large detection range to a variety of gases^23^, have been widely researched in recent decades and are expected to be deployed in domestic, industries, aerospace, and military applications^6^,^24^,^25^,^26^,^27^,^28^,^29^. Tin dioxide (SnO_2_), as an n-type metal oxide semiconductor, has been widely researched for the application in gas sensors with different nanostructures^30^,^31^,^32^,^33^,^34^,^35^. However these nano-units are usually screen-printed or brush-coated on the testing electrodes, which intrinsically limits the reliability and repeatability of the sensors produced. For example, the current printing methods yields reliability issues such as inhomogeneous film thickness, nano-unit size, which heavily affects the effectiveness of the sensing film.

The approach of coupling micro platforms and nano- sensing materials has enormous potential to produce gas sensors with high performance in practical applications^36^,^37^,^38^,^39^. Although current researches have applied nanotechnology for the synthesis of sensing materials and micro-electro-mechanical-system (MEMS) technology for the fabrication of microhotplatforms to improve the detection limit to ppm level, some even to ppb level^40^,^41^. Most of the reported nanoscale sensing materials were produced with a “bottom up” approach^42^,^43^: the pore size, porosity and the film thickness cannot be well controlled, resulting in the uncontrollability and unrepeatability of the sensor’s performance. Besides, special setup and equipment, such as AFM and SEM, are required to load nano materials on the micro platforms, yielding low efficiency and high cost in production^36^,^44^. It is still a challenge to fabricate gas sensors with low power consumption, high sensitivity, fast response time, and low cost that can be reliably produced in batch.

The key issue is how to seamlessly integrate micromachined platforms and nanostructured materials and reserve both merits of microhotplatforms and nanomaterials. To solve this problem, not only high performance microhotplatfroms and nanomaterials are necessary, but a new strategy of sensor fabrication process is also needed. And most importantly, wafer-level fabrication of high-performance is in great demand for practical applications.

Here we propose a novel strategy towards the goal of *in-situ* wafer-level fabrication of such high-performance micro/nano gas sensing chips. First, a 4-inch wafer of microhotplatform (MHP), containing platinum heater and interdigital electrodes (IDEs), is fabricated by micromaching process. Then a 4-inch self-organized polystyrene (PS) spheres monolayer floating on the surface of precursor solution is picked up and transferred to the MHP wafer. After annealing, a wafer-scale monolayer of ordered homogenous nanopore array (NPA) is synthesized *in-situ* on the 4-inch MHP wafer. In this case, a great many of MHP-NPA fused micro/nano gas sensing chips are simultaneously fabricated and arranged on the wafer. Different from all existing fabrication process of gas sensors with nanostructured sensitive materials, our approach combines the micromachining process and the nanofabrication process to naturally integrate MHP with NPA in a wafer scale. Importantly, such method can keep the activities of nanostructured sensitive materials and make the advantages of MHP and NPA. Therefore, the micro/nano gas sensing chips can achieve simultaneously low cost, mass production, as well as sensitive (down to ~20 ppb), rapid (down to ~1 s), and low power (down to ~30 mW) detection. This method has demonstrated a practical way of *in-situ* wafer-level fabrication of gas sensing chips, improving the overall performance of micro/nano gas sensors, and may further suggest a guidance for the design, fabrication, and applications of high-performance gas sensors.

## Results

### Sensing mechanism

Since it was first proposed in 1962, tin dioxide (SnO_2_) has been undergone extensive research and development for gas sensors^45^. Due to the high sensitivity, low operating temperature, and low cost, it has become the dominant choice for solid-state gas sensors. As an n-type semiconductor, conductivity of tin dioxide increases in the presence of reducing gases and decrease in the presence of oxidizing gases.

When oxygen from air adsorbed onto the surface of SnO_2_ particle, electrons from the surface of SnO_2_ are transferred to the adsorbed oxygen. Therefore an electron-depleted region, also called the space-charge layer, is formed near the surface of SnO_2_ particle^46^. Depending on the temperature of the sensor, O_2_^−^ was adsorbed at lower temperature (below 175 ^o^C) and O^−^ and O^2−^ are adsorbed at higher temperatures (above 175 ^o^C)^47^,^48^. So the control of working temperature is critical for the performance of gas sensors.

When exposed to a reducing gas (H_2_, CO, C_2_H_5_OH), surface reactions release electrons back to SnO_2_, leading to a decrease in resistance of the space charge layer^46^,^48^. Decreasing the crystallite size can highly improve the sensitivity, however, the small dimensions are difficult to achieve in practical application^46^,^49^. So in a wafer level fabrication, it is more important to control the size rather than to decrease it.

### Micro/nano gas sensors

**Figure 1:**
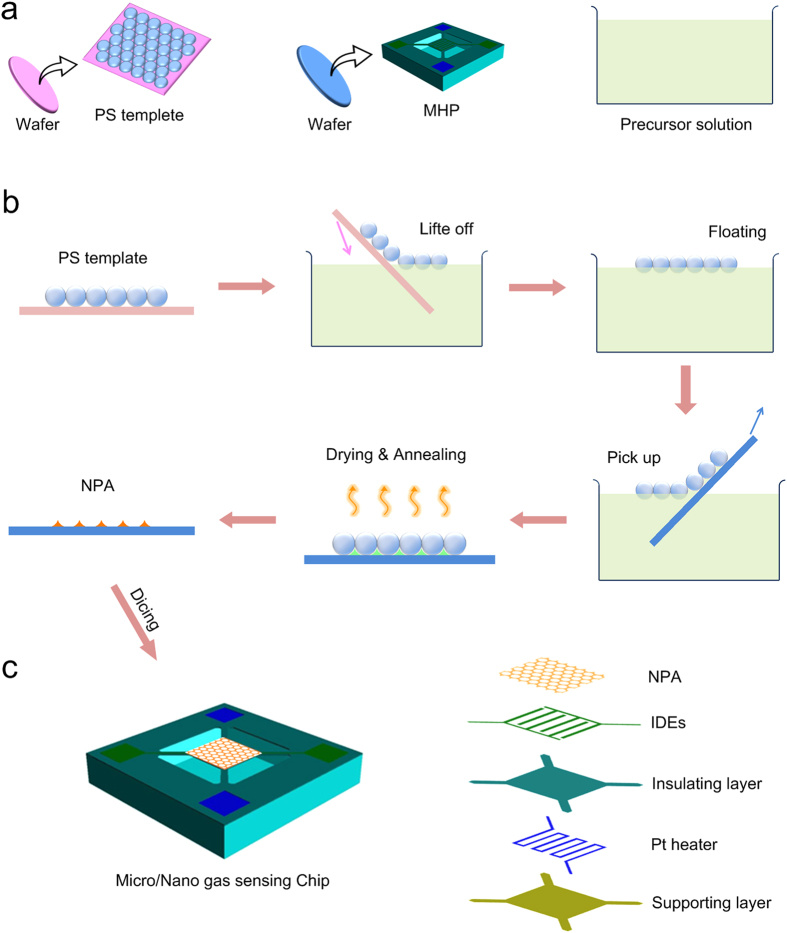
The micro/nano gas sensors and the strategy of *in-situ* wafer-level fabrication process. **** (**a**) For the *in-situ* wafer-level fabrication, a wafer of MHP fabricated by micromachining process, a wafer of polystyrene (PS) colloidal monolayer template, and a precursor solution which is used for synthesis of gas sensing material are respectively prepared in advanced. (**b**) *In-situ* synthesis of SnO_2_ NPA on the MHP. With a precursor solution of SnCl_4_, SnO_2_ can be obtained via hydrothermal methods in basic aqueous solution: Sn^4+^+4OH^−^→SnO_2_+2H_2_O. First, A self-organized PS spheres monolayer template is lifted off from a glass wafer and then floating on the surface of precursor solution (SnCl_4_). Then, such floated monolayer is transferred to the MHP wafer by a simple picking-up process. Due to the capillary effect, the PS monolayer on the wafer also contains the precursor solution in the interstitials among PS spheres and wafer. Along with subsequent drying and annealing, the organic PS template can be removed and ordered SnO_2_ NPA is thus formed on the MHP wafer. In this case, a great many MHP-NPA integrated sensors are simultaneously fabricated and arranged on the wafer. (**c**) Sensor chip and the active area of the micro/nano gas sensor. After the *in-situ* wafer-level fabrication process, the wafer is diced into massive single micro/nano gas sensing chips. The active area of the micro/nano gas sensing chip consists of five layers from bottom to up: supporting layer, Pt microheater, isolation layer, IDEs, and NPA.

Figure 1 illustrates the device design and fabrication process of the micro/nano gas sensor. Based on a silicon substrate, the active area of the micro/nano gas sensing chip consists of five layers from bottom to up: supporting layer, Pt microheater, isolation layer, IDEs, and NPA (Fig. 1c). The detailed fabrication process and photograph of the MHP for micro/nano gas sensors are shown in Figure S1. The square supporting layer, which is made of SiO_2_/Si_3_N_4_, is suspended by four slender supporting beams for thermal isolation. Pt microheater, which is of high mechanical strength and thermal stability, maintains proper working temperature for the sensor with electric current flows through it. With an isolation layer (SiO_2_ or Si_3_N_4_), the micro-spaced IDEs and NPA are well separated with Pt microheater. The resistance of NPA is measured and monitored by IDEs during test. On the silicon substrate, there are four pads, two of which are for injecting current to the Pt microheater. The other two are for measuring the resistance change of gas sensing material.

### *In-situ* wafer-level fabrication process

The *in-situ* wafer-level fabrication process of micro/nano gas sensing chips is to fully combine micromachining process and nanofabrication process and naturally integrate MHP with NPA. As shown in Fig. 1a, a wafer of MHP fabricated by micromachining process, a wafer of polystyrene (PS) colloidal monolayer template, and a precursor solution for synthesis of gas sensing material are respectively prepared in advanced. The template-directed ordered nanopore arrays will exhibit good advantages in the controllable microstructure, homogenous thickness and reproducible fabrication, which is beneficial to fabricate high-performance sensors (Figure S2).

The fabrication process is demonstrated in Fig. 1b. Tin dioxide (SnO_2_), a well-known and wide used gas sensing material, is taken for an example. With a precursor solution of SnCl_4_, SnO_2_ can be obtained via hydrothermal methods in basic aqueous solution:
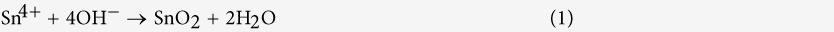


First, A self-organized PS spheres monolayer template^50^,^51^ is lifted off from a glass wafer and then floating on the surface of precursor solution (SnCl_4_). Then, such floated monolayer is transferred to the MHP wafer by a simple picking-up process. Due to the capillary effect, the PS monolayer on the wafer also contains the precursor solution in the interstitials among PS spheres and wafer^52^. Along with subsequent drying and annealing, the organic PS template can be removed and ordered SnO_2_ NPA is thus formed on the MHP wafer. In this case, a great many MHP-NPA integrated sensors are simultaneously fabricated and arranged on the wafer. After dicing, massive single micro/nano sensor chips can be obtained. In our approach, we have two designs of chip size, a normal one (3 mm × 3 mm), and a small one (1 mm × 1 mm) (See Figure S1c–d). Figure S3 shows the operating process of *in-situ* wafer-level fabrication of micro/nano gas sensors. It displays iridescent color (see the central photo in Figure S3) originating from diffraction effect of the thin film, indicating formation of periodic pore arrays on the entire wafer.

All the process in this fabrication method is under well control. The nanostructure, material and thickness of NPA are defined by the size of PS template, material and concentration of the precursor solution. Furthermore, multi-layer NPA can be fabricated by repeating the above procedures. In addition, micro/nano gas sensing chips with different MHP design can also be realized in one batch. Most importantly, such strategy makes a reality of the combination of micro- and nano- fabrication and the integration of MPH with NPA for mass production of gas sensing chips.

### Structure design

**Figure 2:**
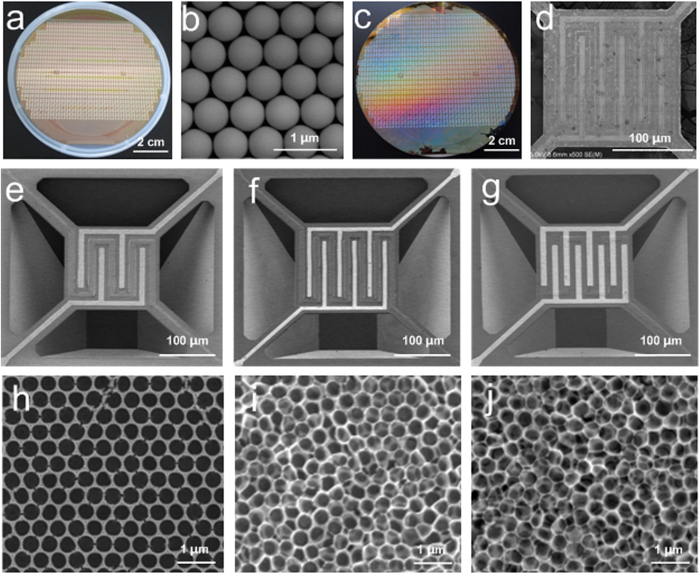
Micro/nano gas sensors. **** (**a**) A 4-inch wafer of MHP, chip size: 3 mm × 3 mm. (**b**) PS template, diameter of the PS ball is 500 nm. (**c**) The whole 4-inch wafer of MHP covered with a monolayer of NPA. (**d**) Active area of the micro/nano gas sensing chip. (**e**) MHP 1, spacing of fingers is 24 μm. (**f**) MHP 2, spacing of fingers is 18 μm. (**g**) MHP 3, spacing of fingers is 10 μm. (**h**) monolayer SnO_2_ NPA. (**i**) double layer SnO_2_ NPA. (**j**) triple layer SnO_2_ NPA.

Figure 2 shows the as-fabricated micro/nano gas sensors. Based on a wafer of MHPs with three different structures and a wafer of PS template with a diameter of 500 nm, micro/nano gas sensors with three kinds of SnO_2_ NPA (monolayer, double layer, and triple layer) have been fabricated respectively. The three MHPs (Fig. 2e–g) have the same Pt microheater (8 μm in width, and 20 μm in spacing), but different IDEs. The spacing of fingers of MHP 1, MHP 2, and MHP 3 are 24 μm, 18 μm, and 10 μm. By using the same PS template and precursor solution, monolayer, double layer, and triple layer SnO_2_ NPA (Fig. 2h–j) are *in-situ* synthesized on the MHP wafers respectively.

This design of MHP has several advantages (Figure S4). (1) Such small active area suspended by four slender beams is of high heating efficiency and hence decrease the power consumption. (2) Heat generated by current flow through Pt microheater can be well isolated in the active area with good temperature uniformity, and the temperature can be precisely controlled by electric power. (3) Due to the thin film structure design, such MHPs are of low thermal mass, which therefore improve the response of sensors: fast in worm-up and cool-down. (4) IDEs with small electrode spacing can decrease the resistance value of sensing material in between two electrodes and hence the Johnson noise^53^.

The SnO_2_ NPA is *in-situ* synthesized on the active area of the gas sensing chips and especially on the IDEs with pores hexagonally arranged due to the geometry of PS template. The SnO_2_ thin film is highly homogenous in morphology and well compatible with the IDEs shown in Fig. 2d. It also shows a good continuity of the NPA at the edges of fingers of IDEs, which indicates that this strategy of fabrication enhances the combination and compatibility of nanostructured gas sensing material and micromachined MHP. In addition, the corresponding phase and microstructural characterization were also addressed in Figure S5.

### Electrothermal characterization

**Figure 3:**
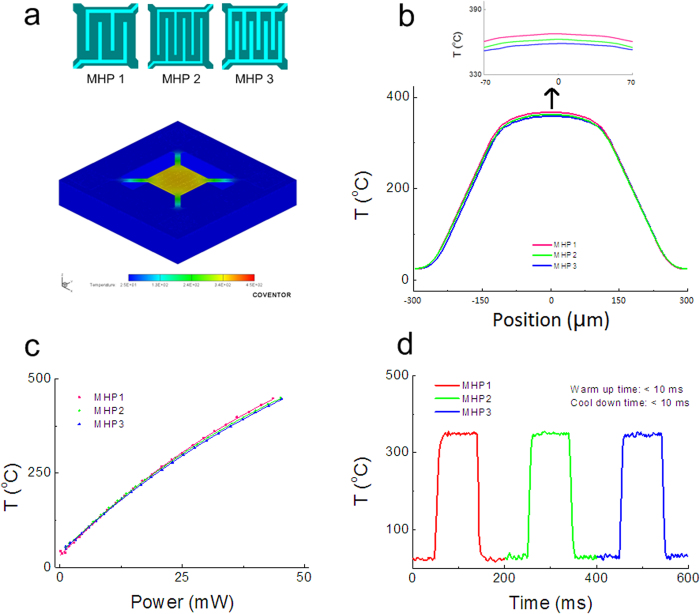
Electrothermal characterization of micro/nano gas sensors. **** (**a**) Temperature distribution on a typical micro/nano gas sensing chip with finite element method (FEM) simulations obtained by commercial analysis software Coventor. Sensors based on MHP 1, MHP 2, and MHP 3 have been introduced in the simulation and comparison. (**b**) Temperature distribution along beam-active area-beam of sensors based on MHP 1, MHP 2, and MHP 3 with a power consumption of 30 mW. The inset is zoom-in of the active area. Temperature gradient on the supporting beams is high, which means that a lot of heat flows through the beams from the heated membrane (active area) to the substrate. The active area achieves a homogenous temperature distribution with an average temperature gradient of about 0.14 ^o^C/μm. The temperature distribution at the active area is generally uniform. Thus, the working temperature of the sensor can be well controlled by power consumption. (**c**) The average temperature of the sensor versus power consumption. It can reach up to 350 ^o^C at a power of 30 mW. Temperature of MHP 3 is slightly lower than that of MHP 1 and MHP 2. But no significant difference in maximum temperature was observed among the three MHPs, which is also illustrated in the simulation results shown in **b**. (**d**) The warm-up time and cool-down time of the sensors. By applying an appropriate step voltage and measuring the resistance change of the Pt microheater, the warm-up time and cool-down time can be measured. It takes less than 10 ms to heat the sensor from room temperature up to 350 ^o^C and cool it down back to room temperature.

Since many semiconductor materials have strong temperature-dependent sensitivity and cross-sensitivity^54^, temperature gradient on the active area should be as small as possible. For optimization, we designed three MHPs with different IDEs layouts (MHP 1, MHP 2, and MHP 3). Figure 3a,b show the electro-thermo-mechanical simulations of temperature distribution on a micro/nano gas sensing chip with finite element method (FEM). Temperature distribution on the device has been obtained with an electric power of 30 mW, by employing commercial analysis software Coventor. Temperature gradient on the supporting beams is high, which means that a lot of heat flows through the beams from the heated membrane (active area) to the substrate. The active area achieves a homogenous temperature distribution with an average temperature gradient of about 0.14 ^o^C/μm.

In a microhotplatform, the total heat loss has three parts: Q_conduction_, Q_ambient_, and Q_radiation_. Q_conduction_ describes the heat conduction through the four supporting beams. Q_ambient_ is the heat loss through the ambient air, and Q_radiation_ is the heat loss due to radiation. Q_conduction_ is the main part of heat loss, which is why temperature of the three microhotplatforms is almost the same at the same power consumption. Conductivity of Pt is higher than that of SiO_2_/SiN_x_. If the surface is covered with more Pt electrodes, heat loss via air and radiation should increase. Therefore, the temperature of MHP 3 is slight lower than that of MHP 1 and MHP 2, shown in Fig 3b,c.

The MHP plays an important role in working temperature and stability of gas sensors. Figure 3c shows the average temperature of the sensor versus power consumption. It can reach up to 350 ^o^C at a power of 30 mW, which is 10 times lower than excellent commercial MOS sensors (Figaro TGS2620, 210 mW)^55^. It also indicates that, especially at a high electric power, the MHP with more pairs of fingers reaches lower temperature than those with fewer fingers. The analysis results (Fig. 3b) agree with the test results. Here, the average temperature of the active area in the device is determined by the resistance R of the heater due to temperature dependent the resistance of the Pt resistor (the details shown in S6). From the analysis results shown in Fig. 3a,b, the temperature distribution at the active area is generally uniform. Thus, the working temperature of the sensor can be well controlled by power consumption.

By applying an appropriate step voltage and measuring the resistance change of the Pt microheater, the warm-up time and cool-down time of the sensor have also been conducted. From the test results shown in Fig. 3d, we can see that it takes less than 10 ms to heat the sensor from room temperature up to 350 ^o^C and cool it down back to room temperature. Due to the small thermal mass, warm-up time and cool-down time of such micro/nano sensor is much faster than traditional gas sensors with a ceramic tube. This feature is particularly useful when a larger number of such sensors are in network, where power consumption is critical. The micro/nano gas sensor can be operated in a pulse voltage/current mode for further power saving.

### Sensor response to ethanol

Sensor response to ethanol has been conducted in a custom-built setup (WS-30 A) at a relative humidity of 50%, as schematically illustrated in Figure S6. Sensitivity and the effects of working temperature, MHP structure and layers of NPA on sensitivity have been evaluated in this work.

Sensitivity of the gas sensor is defined as
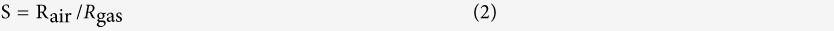
Where R_air_ and R_gas_ are the resistances of the gas sensing material before and after exposure to the target gases, respectively. The resistance of SnO_2_ which is an n-type semiconductor will show an resistance decrease (ΔR) when exposed to ethanol. Then
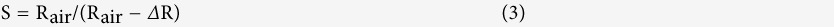

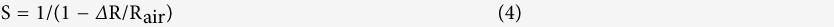


To improve the sensitivity, we can increase the resistance change (ΔR) after exposure to targets gases and decrease the original resistance (R_air_). Nanostructured gas sensing materials with high surface-to-volume ratios, surface activities, and high carrier mobility can effectively improve the resistance change. In our approach, we not only improve ΔR by introducing SnO_2_ NPA as gas sensing material, but decrease R_air_ by MHP with Pt microheater and IDEs.

For an n-type intrinsic semiconductor, the conductivity (G) is determined by G = nqμ_e_, where n, q and μ_e_ are the carrier concentration, elementary charge and the carrier mobility, respectively^56^. We can control the working temperature to change R_air_. Besides, smaller R_air_ can also be achieved just by reducing the spacing in between two fingers.

**Figure 4:**
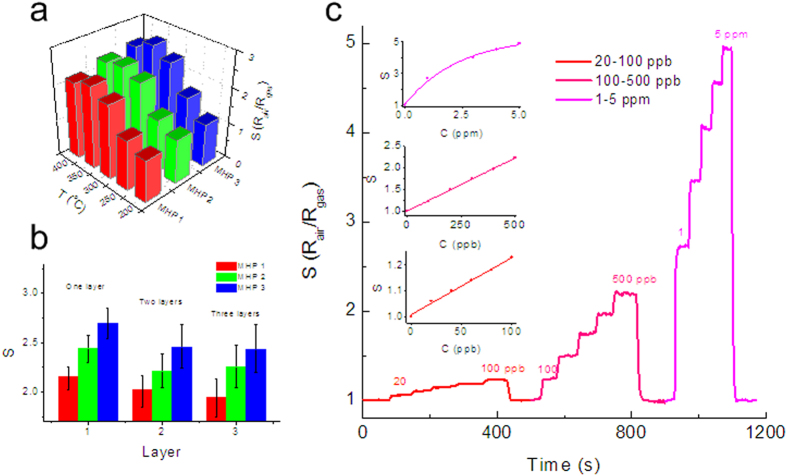
Sensor response to ethanol. **** (**a**) The 3D plot of the sensitivity (S) to 1 ppm ethanol as a function of the working temperature and types of MHP. It clearly indicates that gas sensor has the highest sensitivity at a working temperature of 350 ^o^C. And the sensor based on MHP 3 has higher sensitivity than other two sensors. (**b**) The sensitivities of sensors based on MHP 3, with monolayer, double-layer, and triple-layer of NPA. Sensors with double-layer or triple-layer of NPA have lower sensitivity and larger error than sensors with monolayer of NPA. (**c**) Sensor response to ethanol with three different levels of concentrations: from 20 ppb to 100 ppb, from 100 ppb to 500 ppb, and from 1 ppm to 5 ppm. The response time and recover time to ethanol are around 2 s with a concentration less than 100 ppb, and decrease to around 1 s and less than 1 s when the concentration increase to 100 ppb – 500 ppb and 1 pm – 5 ppm respectively. Sensitivity to 20 ppb ethanol is 1.06. The sensitivity (S) has a linear relation to ethanol concentration (C) via linear fitting can be denote as S = 1 + 0.0023 × C. When the concentration increases to the level of several hundred ppb, sensitivity and concentration still have a good linear relationship as S = 1 + 0.0024 × C. When the concentration of ethanol increase to ppm level, there is a nonlinear relationship between sensitivity and concentration. Sensitivity tends to reach a constant value if the concentration keeps increasing.

Figure 4a shows the 3D plot of the sensitivity (S) to 1 ppm ethanol as a function of the working temperature and types of MHP. It clearly indicates that gas sensor has the highest sensitivity at a working temperature of 350 ^o^C. The sensor based on MHP 3 has higher sensitivity than other two sensors. MHP 3 has narrow gap electrodes that induce a higher collection efficiency of the electrical signal and less influenced by free charges. Besides, MHP 3 has more pairs of fingers that can reduce the contact resistance between Pt and SnO_2_. In addition, nanostructured SnO_2_ has defects such as inactive particles, fractured parts, and SnO_2_ film without nano pores. These defects may be not sensitive or less sensitive to gases.

Based on MHP 3, we then evaluated the properties of sensors with different layers of NPA. Figure 4b shows the sensitivities of sensors with monolayer, double layer, and triple layer SnO_2_ NPA. Sensors with double layer or triple layer NPA have lower sensitivity and larger error than sensors with monolayer NPA. The space charge layer, which is formed near the surface of SnO_2_ particle, can change its resistance when exposed to a reducing gas. Surface reactions release electrons back to SnO_2_, leading to a decrease in resistance of the space charge layer^46^,^48^. In a sensor with multiple layer of SnO_2_, gases can hardly penetrate into the layers beneath the top layer of SnO_2_. Besides, we also found that the quality of the single layer of SnO_2_ is better than that of two or three layers, shown in Fig. 2h–j. Therefore the relative resistance change of a single layer of SnO_2_ is more significant than that of multiple layers of SnO_2_. Sensitivity of a single layer is higher than that of multiple layers. The error comes from fabrication process. The MEMS process of fabricating microhotplatforms has high yield. While in this work, the solution process was conducted by hands, which leads to lower yield and consistency. Based on the results of these test, sensors on MPH 3 with monolayer NPA and working temperature of 350 ^o^C have high sensitivity.

Figure 4c shows the sensor response to ethanol with three different levels of concentrations: from 20 ppb to 100 ppb, from 100 ppb to 500 ppb, and from 1 ppm to 5 ppm. Response time and recover time are defined as the times for the sensor to reach up to 90% of its steady value and back down to 10% of the value^57^, respectively. The response time and recover time to ethanol are around 2 s with a concentration less than 100 ppb, and decrease to around 1 s and less than 1 s when the concentration increase to 100 ppb – 500 ppb and 1 pm – 5 ppm respectively.

It is obvious that the senor is sensitive to 20 ppb ethanol with a sensitivity of 1.06. It indicates that sensitivity (S) has a linear relation to ethanol concentration (C), and their relationship via linear fitting can be denote as
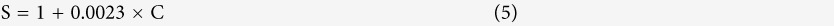


indicating a good linear relation between sensing signals and the concentration. It is acknowledged that a more conductive sensing body may bring a lower Johnson noise and thus heighten the signal-to-noise ratio^58^. In our case, the trace detectable ability may attribute to the higher conductance of micro spaced IDEs and the high-quality NPA.

When the concentration increases to the level of several hundred ppb, sensitivity and concentration still have a good linear relationship as
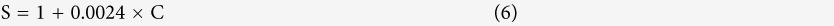


However, when the concentration of ethanol increase to ppm level, there is a nonlinear relationship between sensitivity and concentration. Sensitivity tends to reach a constant value if the concentration keeps increasing. Resistance change (ΔR) of SnO_2_ NPA is the result of ethanol molecule absorbed into the nanofilm. The resistance of SnO_2_ will stop decreasing when enough ethanol molecules absorbed.

Ivanov *et al.* reported Pt-doped SnO_2_ material with a detection limit of 1 ppb ethanol^41^. The sensitivity of tin dioxide could be enhanced by adding Pt and Pd. Our previous work indicated that sensitivity of SnO_2_ sensors based on ceramic tube could by highly improved by adding Pd or Cr^59^. It is speculated that detection limit of the micro/nano sensor could be further improved to sub-ppb level when doped. In comparison with current commercial MOS sensors (based on ceramic tube or thick membrane), this novel micro/nano gas sensor has much higher performance.

Reliability and repeatability has always been the challenge for the real-world applications of micro/nano devices, especially in batch production. This work includes the reliability and repeatability evaluation of the wafer-level micro/nano sensors produced, in terms of consistency of the NPAs, MHPs and the sensing response of each sensing chip. The data in Figures S7-S9 show that the one-batch produced sensors are homogenous in film microstructure, stable in MHP properties and similar in gas sensing response. The performances of power, detection range, and response time are significantly improved by 1 to 2 orders of magnitude.

## Discussion

In this work, we have employed the sacrificial template method to *in-situ* synthesize ordered nanopore array on a 4-inch wafer, aiming to conduct gas sensing chips in a mass-production manner. For remarkable sensitivity to a variety of gases, SnO_2_, an n-type semiconductor, has been indicated as the most promising gas-sensing materials according to considerable investigations. In this study, SnO_2_ was taken as a typical example to demonstrate validity of our *in-situ* wafer-level strategy. The present sacrificial template method can also be easily applied to other materials, as indicated in our previous researches, such as In_2_O_3_,^60^ Fe_2_O_3_^61^, and LaFeO_3_^62^. However, these previous literatures were all based on the high-power (0.5-2 W) alumina substrates. The main focuses in this study are directed at a new strategy towards *in-situ* wafer-level fabrication of gas sensing chips in a mass-production fashion.

In comparison with other strategies of the fabrication of MOS gas sensors with microhotplatforms and nano-sized SnO_2_ as a sensitive material^15^,^16^,^17^,^18^,^31^,^32^,^33^,^35^, our strategy is of lower cost, batch production, and more controllability. Not only the size, sensitive material, and doped catalysts can be well controlled, but the working temperature, sensitivity, and detection limit can be also controlled. The sensitivity and detection limit of the as-fabricated SnO_2_ sensor is in the level of tens of ppb, which is as better as current high-sensitivity gas sensors with nano-sized SnO_2_. To improve the selectivity of gas sensors, catalysts such as Pt^41^, Pd^59^, and Cr^59^ could be easily added during the synthesis of sensing materials based on our solution-dipping template method. More importantly, sensors produced by such wafer-level strategy are homogenous in film microstructure, stable in MHP properties and similar in gas sensing response.

In conclusion, a strategy towards *in-situ* wafer-level fabrication process is presented and evaluated for the fabrication of micro/nano gas sensing chips with high performance and high throughput. The integration of the micromachining process and the nanofabrication process fully preserves the merits of both MHP and NPA to massively produce thousands of gas sensing chips with high sensitivity (down to 20 ppb), low power consumption (down to 30 mW), and fast response time (down to 1 s). It is also demonstrated that such fabrication process yields high reliability and high repeatability, which enables high throughput and low cost production for practical applications.

## Methods

### Fabrication of MHP

The MHP wafer was fabricated based on classic MEMS processes. (i) A double-side-polished N-type <100> oriented silicon wafer with a layer of SiO_2_ (350 nm in thickness) thermally grown at 1100 °C; (ii) Then a Si_3_N_4_ (300 nm in thickness)/ SiO_2_ (200 nm in thickness) membrane was successively deposited on each side of the silicon substrate by low pressure chemical vapor deposition (LPCVD) at 800 °C; (iii) The Pt/Ti electrodes (10 μm wide and 10 μm separated, 200 nm in thickness) and bonding pads were patterned by lift-off process; (iv) An insulating layer of Si_3_N_4_ (400 nm in thickness) was deposited on it by plasma enhanced chemical vapor deposition (PECVD); (v) Then the Pt/Ti interdigital electrodes (10 μm wide and 10 μm spacing, 200 nm in thickness) and leading wires were patterned by lift-off process. (vi) Positive photolithography was used to define the corrosion windows for releasing the heating membrane area and the support cantilever; (vii) Under the protection of the photoresist, the exposed silicon oxide and silicon nitride composite membrane were etched completely using reactive ion etching (RIE); (viii) After that, the whole membrane was released by wet chemical anisotropic etching using a solution of TMAH (25 wt.%) at 80 °C.

### Preparation of PS monolayer template

Si wafer (4 inch), with 0.5 mm in thickness, were cleaned according to the procedures reported previously^51^. Suspension of monodispersed PS with 500 nm in diameter (2.5 wt % in water) was purchased from Alfa Aesar Corporation. The PS colloidal monolayer template was prepared on the well-cleaned Si wafer by air/water interfacial assembly. In brief, the PS sphere suspensions were diluted in the same volume ethanol and ultrasonicated for absolute uniformity. The mixed suspension was slowly injected into the edge of water-film covered Si wafer with a micropipette for about 10 min. After injection, we kept it for 5 min for PS spheres’ self-assembly at the air/water interface. Finally, the large-area PS sphere monolayer was formed on the wafer after liquid evaporation by blowing gentle airflow at 30 °C.

### Wafer-level fabrication

*In-situ* wafer-level fabrication process of sensor chips is based on a sacrificial template method with solution dipping, as illustrated in Fig. 1. Firstly, a Si wafer (4 inch) covered by PS sphere colloidal monolayer template, with the sphere diameter of 500 nm, was integrally lifted off by aslant dipping into a 0.1 M SnCl_4_ precursor solution in a beaker due to surface tension of the solution and then floated on the solution surface. In succession, the floating PS colloidal monolayer was picked up with the MHP wafer (4 inch) and dried at 120 °C for 0.5 h. At this stage, owing to the strong hydrolysis of Sn^4+,^ 63, there must undergo the hydrolysis reaction and it mainly produces Sn(OH)_4_ (or H_2_SnO_3_•H_2_O). After it was subsequently heated at 400 °C for 2 h, the PS template was burned away, a wafer-scaled ordered porous SnO_2_ thin film was formed on the MHP wafer, and thus a great many MHP-NPA integrated sensors are simultaneously fabricated and arranged on the wafer.

### Structure characterization

Figure S5 Shows the structural characterization of nano-sized SnO_2_. Figure S5a gives the cross-sectional SEM of the as-synthesized nano pore array. The size of the hole is 500 nm. Figure S5b gives the X-ray diffraction (XRD) patterns for the as-synthesized SnO_2_. The peaks the sample are well matched with standard PDF card of SnO_2_ (No. 41-1445), indicating a phase of tetragonal rutile. Further, the microstructure was examined. Figure S5c shows the transmission electron microscopic (TEM) image and the corresponding selected-area electron diffraction (SAED) pattern (inset) of SnO_2_. The grain size is smaller than 5 nm (as marked with the dot-line in Figure S5c). Additionally, the corresponding SAED pattern has demonstrated that it is polycrystalline SnO_2_.

### FEM simulation

FEM simulations have been done by using the electro-thermo-mechanical simulations of commercial analysis software Coventor. Simulations have been performed assuming the following boundary conditions: (a) The temperature on the back side of the die is constant and set as room temperature 25 ^o^C; (b) On the upper and lower surfaces of the membrane, heat is dissipated through convection and radiation; (c) Electric voltages are applied on the pads of the Pt heater. Some parameters of thin films are different from those in bulk materials. Thermal conductivities used in our simulations are 73, 22, 1.4 157, 0.026 W/mK for platinum, silicon nitride, silicon dioxide, silicon and air, respectively.

### Temperature of MHP

Temperature of MHP is calculated by T = (R − R_o_)/(α R_o_) + 25, which is widely used to extract the average temperature of the active area in gas sensing applications. where α is the temperature coefficient of resistance (TCR) of platinum, R is the measured resistance, R_o_ is the original resistance at room temperature (25 ^o^C), and T is the average temperature of the active area. By measuring the resistance change, average temperature can be calculated.

## Additional Information

**How to cite this article**: Xu, L. *et al*. Micro/Nano Gas Sensors: A New Strategy Towards *In-Situ* Wafer-Level Fabrication of High-Performance Gas Sensing Chips. *Sci. Rep.* **5**, 10507; doi: 10.1038/srep10507 (2015).

## Supplementary Material

Supporting Information Supplementary Figures 1-6
