# Supplementary material for: Micro/Nano Gas Sensors: A New Strategy Towards In-Situ Wafer-Level Fabrication of High-Performance Gas Sensing Chips
Source: Sci Rep. 2015 May 22;5:10507. doi: 10.1038/srep10507 (PMC5377049; doi:10.1038/srep10507)
Supplement: Supporting Information — Supplementary Figures 1-6 [file srep10507-s1.doc]

**Supporting Information**

**Micro/Nano Gas Sensors: A New Strategy Towards In-Situ Wafer-Level Fabrication of High-Performance Gas Sensing Chips**

*Lei Xu^1,3,*^, Zhengfei Dai^2,*^, Guotao Duan^2^, Lianfeng Guo^1^, Yi Wang^1^, Hong Zhou^1^, Yanxiang Liu^1^, Weiping Cai^2^, Yuelin Wang^1^, and Tie Li^1^,*

1 State Key Laboratory of Transducer Technology, and the Science and Technology on Micro-system Laboratory, Shanghai Institute of Microsystem and Information Technology, Chinese Academy of Sciences, Shanghai, 200050, China.

2 Key Lab of Materials Physics, Anhui Key lab of Nanomaterials and Nanotechnology, Institute of Solid State Physics, Chinese Academy of Sciences, Hefei, 230031, Anhui, China.

3 California Institute of Technology, Pasadena, California 91125, USA.

* These authors contributed equally to this work.

Correspondence and requests for materials should be addressed to T. L. (tli@mail.sim.ac.cn) or to G. D. (duangt@issp.ac.cn).

**S1.** **Fabrication of MHP Wafer**

The MHP wafer was fabricated based on classic MEMS processes, shown in Figure S1 a. (i) A double-side-polished N-type <100> oriented silicon wafer with a layer of SiO_2_ (350 nm in thickness) thermally grown at 1100 ºC; (ii) Then a SiN_x_ (300 nm in thickness)/ SiO_2_ (200 nm in thickness) membrane was successively deposited on each side of the silicon substrate by low pressure chemical vapor deposition (LPCVD) at 800 ºC; (iii) The Pt/Ti electrodes (10 μm wide and 10 μm separated, 200 nm in thickness) and bonding pads were patterned by lift-off process; (iv) An insulating layer of SiNx (400 nm in thickness) was deposited on it by plasma enhanced chemical vapor deposition (PECVD); (v) Then the Pt/Ti interdigital electrodes (10 μm wide and 10 μm spacing, 200 nm in thickness) and leading wires were patterned by lift-off process. (vi) Positive photolithography was used to define the corrosion windows for releasing the heating membrane area and the support cantilever; Under the protection of the photoresist, the exposed silicon oxide and silicon nitride composite membrane were etched completely using reactive ion etching (RIE); After that, the whole membrane was released by wet chemical anisotropic etching using a solution of TMAH (25 wt.%) at 80 ºC. The active area of MHP is shown in Figure S1 b. And two wafers of MHPs with different chip size are shown in Figure S1 c-d.


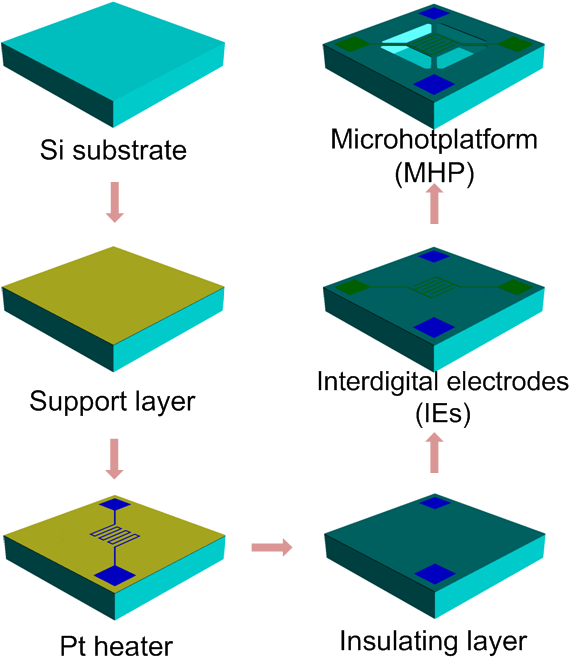

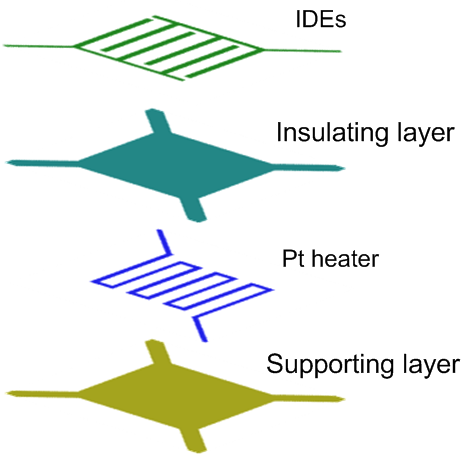

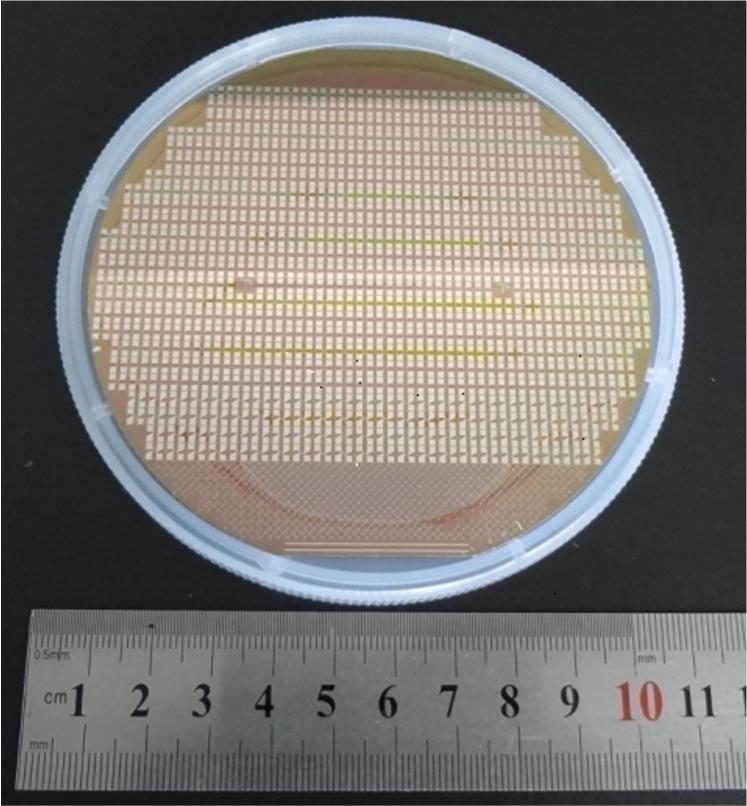

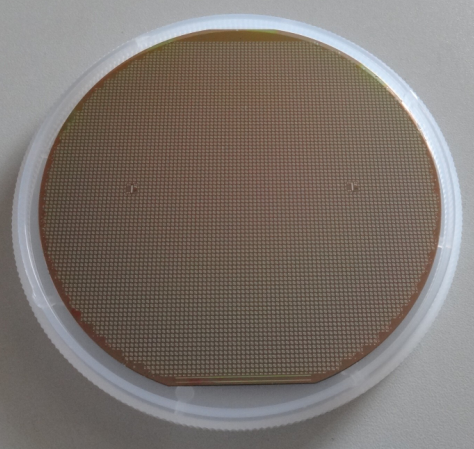


(a)

(b)

(c)

(d)

**Figure S1.** Fabrication of MHP. (a) fabrication process, (b) explosive view of the active area of MHP, (c) chip size: 3 mm × 3 mm, (d) chip size: 1 mm × 1 mm.

**S2. Structure design of the nanopore array**

Sensitivity of MOS sensors is proportional to the relative change of resistance of the sensitive material when exposed to target gases. Most of current sensitive materials are made of unordered nanofilm which is shown in Figure S2 a. Different grain sizes, active facets, and barriers lead to different resistances and resistance changes, which therefore influence the sensitivity of the sensor. To improve the sensitivity, we designed a highly ordered nanopore array, which is formed by a sphere template, as shown in Figure S2 b. The advantage of the ordered nanopore array is to improve the homogeneity of the gas sensing material. Consequently, sensitivity can by highly improved and controlled.


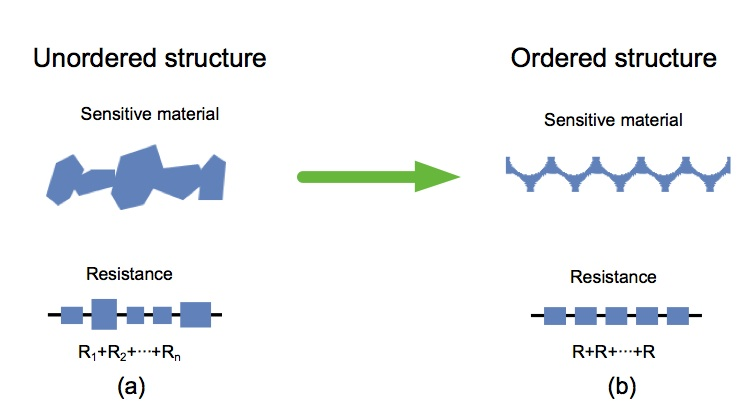


**Figure S2**. Comparison of gas sensing materials with unordered structure (a) and ordered structure (b).

**S3.** **In-situ wafer-level fabrication of micro/nano gas sensors**

The SnO_2_ ordered porous thin-ﬁlm gas sensing devices were fabricated by transferring the solution-dipped self-organized PS colloidal template onto the above chip. Firstly, an ordered PS colloidal monolayer template, with the sphere diameter of 500 nm, was prepared by air/water interfacial assembly. Such a monolayer on the glass substrate was integrally lifted off by aslant dipping into a 0.1 M SnCl_4_ precursor solution in a beaker due to surface tension of the solution and then ﬂoated on the solution surface. In succession, the floating PS colloidal monolayer was picked up with the UV-cleaned MHP wafer and dried at 120 °C for 0.5 h. After it was subsequently heated at 400 °C for 2 h, the PS template was burned away, an ordered porous SnO_2_ thin ﬁlm was formed on the wafer, and thus the gas sensors, integrating micro/nanostructured porous thin film with MHP chips, were one-batch fabricated. Figure S3 shows strategy of In-situ wafer-level fabrication process.


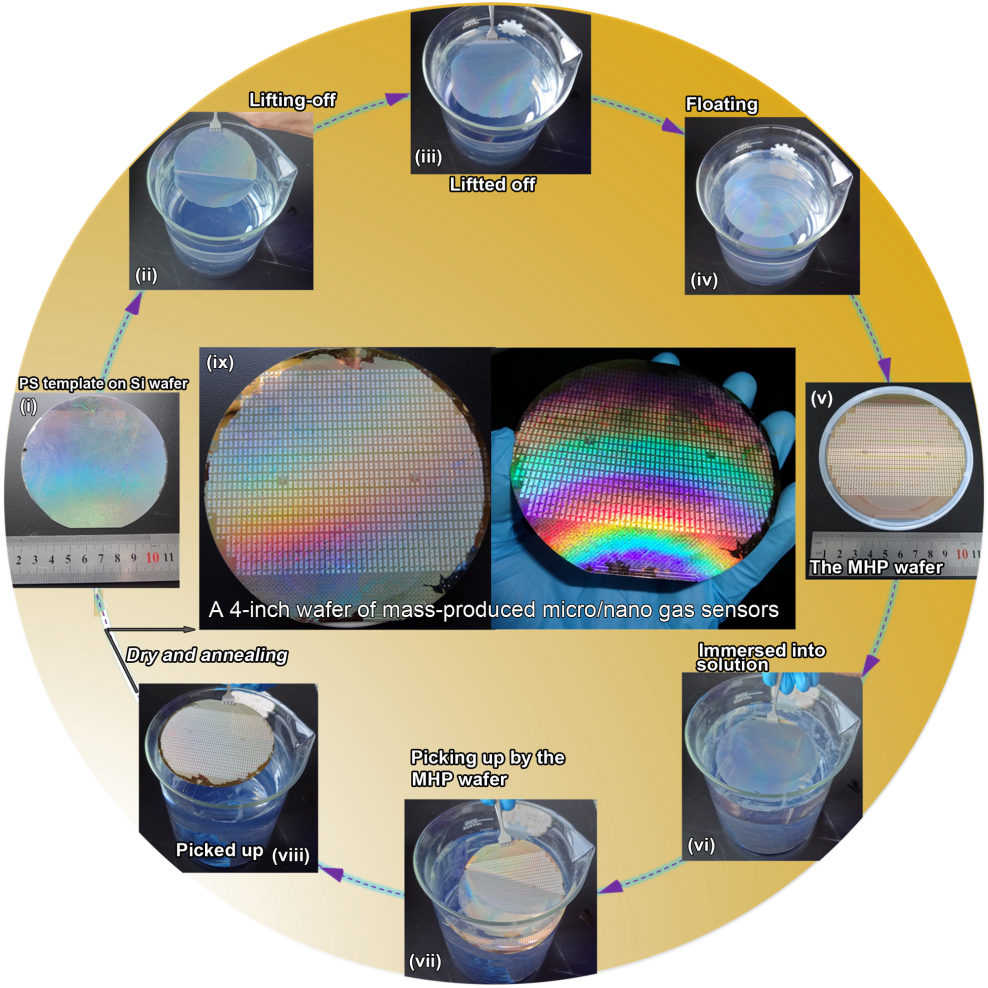


**Figure S3.** The in-situ wafer-level fabrication of micro/nano gas sensors.

**S4 Microhotplatform for maintaining working temperature**

Sensitivity of MOS sensors is dramatically influenced by the working temperature. Conventional MOS sensors usually have a ceramic tube with a Pt coil in it to supply heat for the sensor. However temperature homogeneity of the ceramic is very low, with a high temperature in the center of the tube, as shown in Figure S4 a. Therefore, it is almost impossible to control all the gas sensing material working at the same state by changing power supply. So how to design a structure to make the whole film of sensing material at the same working temperature is a challenge.

In order to improve the performance, we designed a suspended membrane type microhotplatform for the gas sensors, shown in Figure S4 b. Compared to conventional ceramic tube, this structure offers at least two advantages to improve the sensitivity: (i) working temperature of the sensor can be well controlled by applying appropriate voltages on the Pt heater; (ii) due to the design, temperature distribution on the active area has better homogeneity. Sensitivity of the sensor can by improved (or controlled) by changing the electric power.


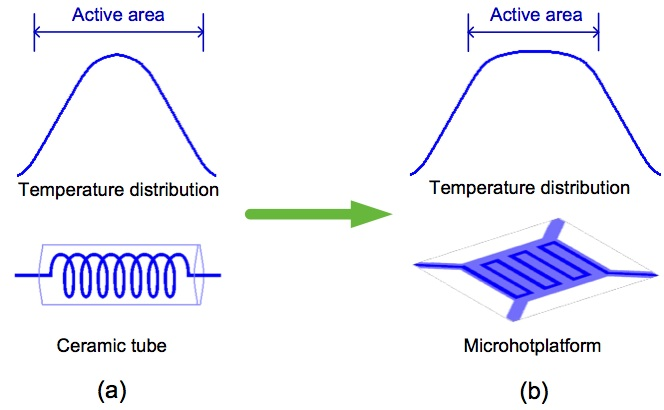


**Figure S4**. Comparison of temperature distribution on the active area of a ceramic tube (a) and a microhotplatform (b).

**S5. FEM simulation**

Finite Element Method (FEM) simulations have been done by using the electro-thermo-mechanical simulations of commercial analysis software Coventor. Simulations have been performed assuming the following boundary conditions: (a) the temperature on the back side of the die is constant and set as room temperature 25 ^o^C; (b) on the upper and lower surfaces of the membrane, heat is dissipated through convection and radiation; (c) electric voltages are applied on the pads of the Pt heater.

Some parameters of thin films are different from those in bulk materials. The parameters used in our simulations are listed in Table S1.

**Table S1**. Parameters used in the FEM simulation

| **Materials** | **Thermal conductivity**  **(W/mK)** | **Thermal expansion**  **(1/K)** | **Density**  **(Kg/m^3^)** | **Heat capacity**  **(J/kgK)** |
| --- | --- | --- | --- | --- |
| Pt | 73 | 8.9×10^-6^ | 2.145×10^4^ | 130 |
| Si_3_N_4_ | 22 | 2.33×10^-6^ | 3.1×10^4^ | 700 |
| SiO_2_ | 1.4 | 0.55×10^-6^ | 2.2×10^3^ | 730 |
| Si | 157 | 2.33×10^-6^ | 2.32×10^3^ | 700 |
| air | 0.026 | - | 1.16 | 1000 |

**S6. Structural characterization**

Figure S5 Shows the phase and structural characterization of nano-sized SnO_2_. Figure S5 a gives the cross-sectional SEM of the as-synthesized nano pore array. The size of the hole is 500 nm. Figure S5 b gives the X-ray diffraction (XRD) patterns for the as-synthesized SnO_2_. The peaks of the sample are well matched with standard PDF card of SnO_2_ (No. 41-1445), indicating a phase of tetragonal rutile. Further, the microstructure was examined. Figure S5 c shows the transmission electron microscopic (TEM) image and the corresponding selected-area electron diffraction (SAED) pattern (inset) of SnO_2_. The grain size is smaller than 5 nm (as marked with the circle dot-line). Additionally, the corresponding SAED pattern has demonstrated that it is polycrystalline SnO_2_.


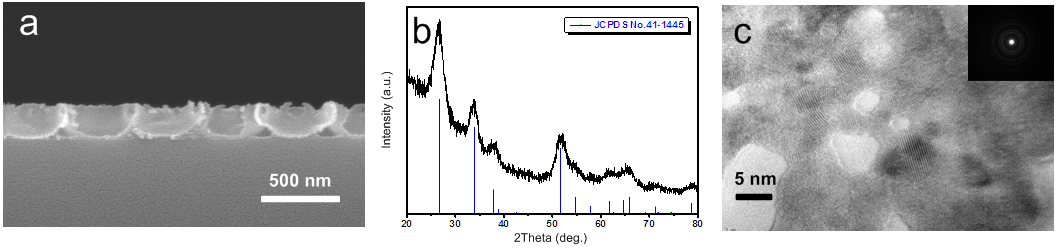


**Figure S5.** Structural characterization of nano-sized SnO_2_. (a) Cross-sectional SEM of the as-synthesized nano pore array; (b) XRD spectra of SnO_2_. (c) TEM image and the corresponding SAED pattern (inset) of SnO_2_.

**S7. Temperature extraction**

Electrical characteristics of the sensor have been tested. Temperature was calculated by equation (1) which is widely used to extract the average temperature of the active area in gas sensing applications.

T=(R-R_o_)/(α R_o_)+25 (1)

where α is the temperature coefficient of resistance (TCR) of Pt, R is the measured resistance, R_o_ is the original resistance at room temperature (25^o^C), and T is the average temperature of the active area in ^o^C.

By measuring the resistance change, average temperature can be calculated by equation (1).

**S8. Measurement of gas sensing performances**

Gas-sensing experiments were performed in a static system in a custom-built experiment setup (WS-30A) at relative humidity 60% and 25 °C. It should be mentioned that there are two small mixing fans mounted in the sample chamber to mix the test gases sufficiently in very short time. A certain amount of gas or volatile liquid was injected into the gas chamber with a sample in the system. The gas concentration in the chamber could be determined based on the injected amount. The sensing response was obtained by measuring the change of the electrical voltage of the sensing devices. Figure S6 shows the schematic illustration of the gas sensing process in a static testing system.


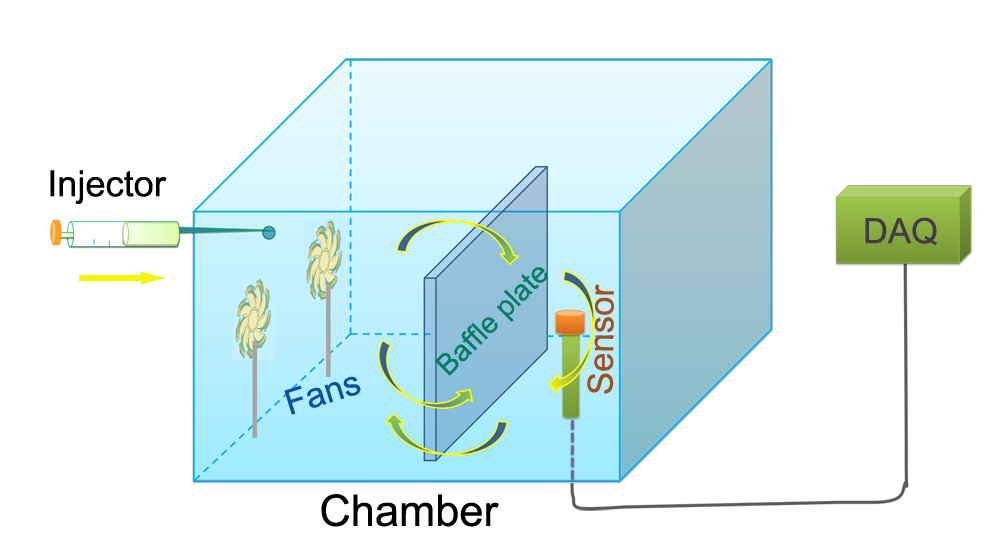


**Figure S6.** A schematic illustration of the gas sensing process in a static testing system.

**S9. Consistency of the micro/nano gas sensors in a wafer scale**

By the micro/nano integrated process, gas sensors can be mass produced in batches. Consistency of the micro/nano gas sensors should also be evaluated in a wafer scale. Figure S7, Figure S8 and Figure S9 show the performances of the mciro/nano gas sensors fabricated by the same procedure.


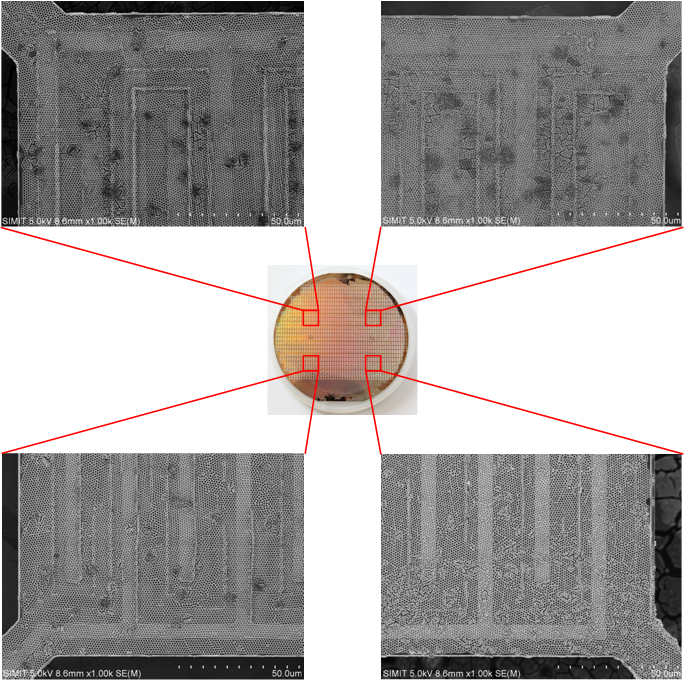


**Figure S7**. SEM images of micro/nano gas sensors at different locations of the wafer.


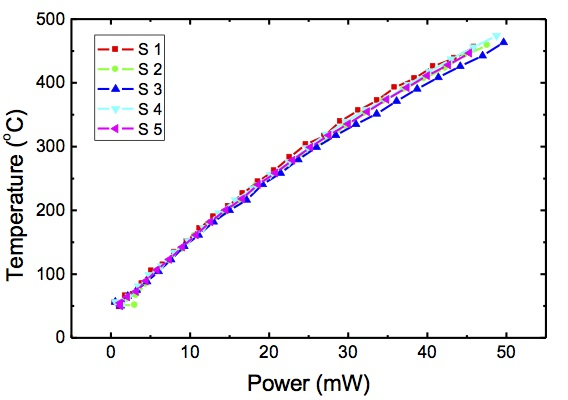


**Figure S8**. Temperature versus power of five samples (based on MHP 3) from different locations of one Si wafer.


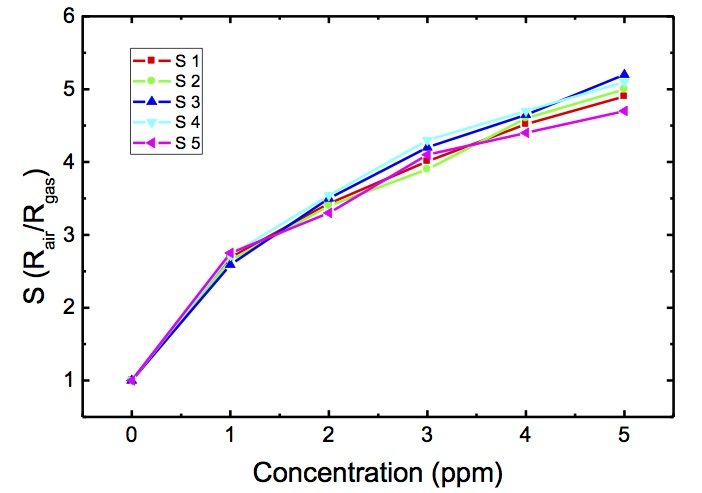


**Figure S9**. Sensitivities of five samples (MHP 3, monolayer SnO_2_ NPA, Ethanol) from different locations of the wafer.
